# Teeth Discolored by Arsenious Acid

**Published:** 1850-01

**Authors:** A. Berry

**Affiliations:** Raymond, Miss.


For the Dental Register of the West

### TEETH DISCOLORED BY ARSENIOUS ACID.

BY A. BERRY, D. D. S.—*Raymond, Miss.*

About six years ago, I had as a patient, a lady of this place, with the superior lateral incisors so badly decayed, on their palatal surfaces, near their necks, that at first I refused to fill them; but, yielding to the urgent solicitations of the lady and her friends, I consented to make an effort to save them for a few years. Finding the decay to extend nearly to the nerve cavities, after excavating it in part, I applied the common arsenical preparation to remove an excessive tenderness, and ordered the patient to displace it, after the lapse of a certain period. I called to see her *thirty hours afterwards*, and found that my directions had been disregarded, and the preparation still secure in the cavities; and saw with deep chagrin, that the color of the crowns of the teeth had been changed to a purple hue. I finished the operation of filling them; *but have never put arsenious acid in a front tooth since*, having generally used extract of nut gall in similar cases.

There has been no advance of decay in these teeth to the present time. The one least diseased has almost fully recovered its former color, while the other has become darker than at the time of filling it.

